# Next5 – A new South African Academy of Family Physicians initiative (‘You didn’t come this far, to only come this far’)

**DOI:** 10.4102/safp.v63i1.5405

**Published:** 2021-10-26

**Authors:** Chantelle Van der Bijl, Arun Nair, Klaus B. von Pressentin

**Affiliations:** 1 Department of Family Medicine, Faculty of Health Sciences, University of the Free State, Bloemfontein, South Africa; 2 Robert Mangaliso Sobukwe Hospital, Northern Cape Department of Health, Kimberley, South Africa; 3 Division of Family Medicine, School of Public Health and Family Medicine, Faculty of Health Sciences, University of Cape Town, Cape Town, South Africa

Interviews with district managers to understand the impact of family physicians (FPs) on the South African health system confirmed several benefits, including their ability to increase access to a more comprehensive and coordinated health service, to improve clinical care, to capacitate the healthcare team and facilitate clinical governance activities.^1^ However, the FP’s leadership abilities and capacity to influence their team was seen as a key factor in determining their impact on health outcomes. The study highlighted that the integration of new FPs into the healthcare team requires active management, including the need for role clarification and supporting role maturation over time.

**FIGURE 1:**
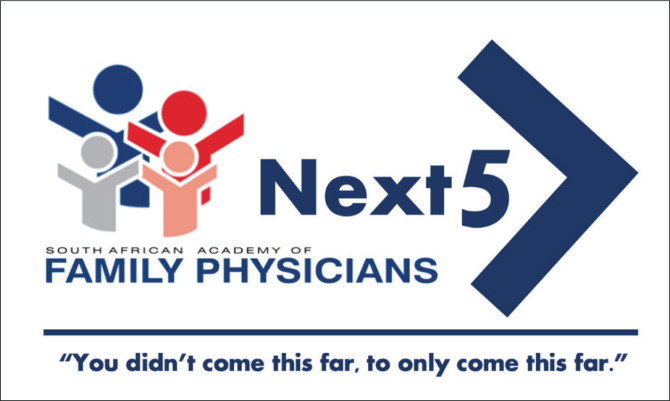
The Next5 logo and slogan.

At the April 2021 meeting of the South African Academy of Family Physicians education and training committee (ETC), it was agreed to set up a working group to look at an initiative on how the academy can assist newly qualified FPs within their first 5 years of qualifying, similar to the First five concept of the Royal College of General Practitioners and the Young Doctors Movements of WONCA (World Organization of Family Doctors).^2,3^ A survey was held amongst South African Academy of Family Physicians (SAAFPs) members to explore the needs and suggestions for activities for this special interest group, which is named Next5 (see Figure 1, which depicts the logo and slogan of this group, as approved by the ETC). Between 23 July 2021 and 31 July 2021, all family medicine registrars and qualified FPs were invited to complete an online survey, which aimed to explore how Next5 can assist in empowering newly qualified FPs and to determine the interest in joining Next5 and its planned activities. The findings of the survey were presented at the August 2021 ETC meeting. A total of 77 responses were received with 28.9% from the target group (FPs qualified in the past 5 years), 10.5% from FPs qualified 5–10-years ago and 38.2% from FPs qualified more than 10-years ago. The remaining responses were from registrars, with the majority in their 3rd and 4th year of training. Table 1 presents Next5 activities as suggested by the survey respondents. Encouragingly, 38 respondents indicated that they would like to join Next5 and 25 senior FPs were keen to serve as mentors to FPs in the first 5 years of their career.

**TABLE 1:** Activity suggestions for the Next5 special interest group as informed by the membership survey.

Activity category	Activity examples
Communication and marketing activities	Creating and maintaining a database of FPs in their first 5-years, as well as maintaining a database of current and potential mentors. Dedicated area on SAAFP website and other platforms, such as WhatsApp/Telegram. A possible section in the SAFP journal, ‘Transition tips’, aimed at Next5 members.
Graduation, registration and career guidance initiatives	Welcome/graduation package for newly qualified FPs, including how to register with the HPCSA as a FP specialist and orientation to SAAFP full membership benefits. Orientation to possible FP career options: clinical/research/teaching (and how to grow as clinician-educators/researchers/scholars). Assist with preparation for consultant interviews. Assist with transition to private sector: involve private sector FPs for mentorship, practice and staff management and how to navigate medical aids, coding and billing.
Mentoring and networking activities	Mentorship by senior FPs, including a mentoring network, which capacitates FPs to serve as mentors in the Next5 group. Connect with and strengthen existing local FP forums and activities. Assist SAAFP and ETC to develop activities aimed at Next5 members. Linking with WONCA World and WONCA Africa Young Doctors’ initiatives.

FP, family physicians; SAAFP, South African Academy of Family Physicians; HPCSA, Health Professions Council of South Africa; ETC, education and training committee; WONCA, World Organization of Family Doctors.

As part of the August 2021 ETC presentation, we reviewed the number of registrars who became SAAFP members after graduation (Table 2). Reassuringly, the numbers over the past 2 years have improved, but there appears to be an unmet need in this group of potential SAAFP members. Newly appointed FPs need an active process of support from and role clarification within their healthcare teams to establish themselves in the health system and to mature in all their different roles.^1^ By encouraging existing FPs to support new FPs in their transition from registrar to expert generalist trained to work in both public and private healthcare sectors, we trust that this new Academy initiative will assist with creating an ongoing ‘sense of belonging’ in the SAAFP community and tangible membership benefits for all newly qualified FPs. We encourage all registrars to continue their full membership of the SAAFP after graduation and become involved with the Next5 activities. If you are keen to learn more about this initiative and wish to become involved, please email to admin@saafp.org.

**TABLE 2:** National overview of registrars who became South African Academy of Family Physicians members after graduation.

Group	2015	2016	2017	2018	2019	2020	Total
Total registrars	180	159	161	170	196	165	1031
Graduates	23	21	34	26	12	18	134
Number of graduates who became SAAFP members	2	11	5	5	9	15	47
Percentage of graduates who became SAAFP members	8.7	52.4	14.7	19.2	75.0	83.3	35.1

*Source:* South African Academy of Family Physicians (used with permission)

SAAFP, South African Academy of Family Physicians.
